# *BDNF* rs10501087, rs1491850 and rs11030094 polymorphisms associated with delayed progression in early-stage Parkinson's disease

**DOI:** 10.3389/fneur.2022.1053591

**Published:** 2022-11-17

**Authors:** D. Luke Fischer, Peggy Auinger, John L. Goudreau, Katrina L. Paumier, Allyson Cole-Strauss, Christopher J. Kemp, Jack W. Lipton, Caryl E. Sortwell

**Affiliations:** ^1^Department of Translational Neuroscience, College of Human Medicine, Michigan State University, Grand Rapids, MI, United States; ^2^Department of Neurology, Center for Health and Technology, University of Rochester, Rochester, NY, United States; ^3^Department of Neurology and Ophthalmology, College of Osteopathic Medicine, Michigan State University, East Lansing, MI, United States; ^4^Hauenstein Neuroscience Center, Mercy Health Saint Mary's, Grand Rapids, MI, United States

**Keywords:** Parkinson's disease, brain-derived neurotrophic factor, rs908867, rs11030094, rs10501087, rs1157659, rs1491850

## Abstract

Parkinson's disease (PD) is heterogenous in its presentation, progression and response to therapies. Genetic polymorphisms may account for some of this variability. Several single nucleotide polymorphisms (SNPs) in the brain-derived neurotrophic factor gene *BDNF* have been associated with differing clinical outcomes from different dopaminergic replacement strategies, and one of these, the rs6265 SNP, has been associated with a milder clinical phenotype in the unmedicated, early-stage of PD. We examined if other *BDNF* SNPs with potential pharmacogenetic effects also are associated with different rates of disease progression. The Deprenyl And Tocopherol Antioxidative Therapy Of Parkinsonism (DATATOP) study was analyzed retrospectively. DNA samples (*n* = 217) were genotyped for the *BDNF* rs908867, rs11030094, rs10501087, rs1157659, and rs1491850 SNPs, and the primary endpoint was time to initiate symptomatic pharmacotherapy. Genotypes were compared using the Cox proportional hazard ratio (HR) with baseline age, sex, site, time since PD diagnosis and rs6265 genotype as covariates. The primary endpoint was associated with a delay with three SNPs: rs10501087 [HR (95% Confidence Interval) = 28.3 (3.6–223.1, *p* = 0.002) and 7.6 (1.9–29.8, *p* = 0.004) for T/T and T/C subjects, respectively, vs. C/C subjects], rs1491850 [HR = 3.3 (1.3–8.4, *p* = 0.04) and 2.8 (1.3–6.4, *p* = 0.03) for T/T and T/C subjects, respectively, vs. C/C subjects] and rs11030094 [HR = 2.5 (1.1–5.6, *p* = 0.03) and 2.0 (1.3–6.4, *p* = 0.03) for A/A and A/G subjects, respectively, vs. G/G subjects]. From the primary endpoint, specific rs10501087, rs1491850, and rs11030094 SNP genotypes are associated with a slower rate of PD progression in the unmedicated state. A prospective clinical trial examining many *BDNF* SNPs is warranted.

## Introduction

There is considerable heterogeneity in the rate of progression of Parkinson's disease (PD) as well as in a patient's presenting phenotype and clinical outcomes with therapies (1). Underlying disease variability likely is explained, at least partly, by genetic variation. Gene variants can influence risk for developing PD and exert pharmacogenetic effects (2, 3), and these variants may have independent yet related effects on PD risk, phenotype, progression and therapeutic response (4–6). Disentangling genetic effects on progression from the confound of ongoing pharmacotherapy on clinical endpoints has been difficult, given the paucity of rigorous studies that enrolled early-stage, PD subjects not requiring medical therapy. In addition, prior investigations have focused more on genes linked to increased incidence of PD.

Recent reports have implicated the gene *BDNF*, which codes for brain-derived neurotrophic factor (BDNF), in several facets of PD heterogeneity. In particular, the *BDNF* rs6265 single nucleotide polymorphism (SNP), which is present in ~35% of the population (7), has been associated with variable disease progression (8, 9), though not increased incidence (10). Further, the rs6265 SNP has been associated with poorer clinical outcomes in early-stage PD subjects who are treated with levodopa monotherapy (6, 11), and this SNP may be associated with earlier development of levodopa-induced dyskinesias (12, 13). Whereas the rs6265 SNP has received the most attention of those reported in the gene *BDNF*, others are associated with modest hippocampal or whole-brain atrophy, cognitive dysfunction and with potential pharmacogenetic effects on dopamine replacement strategies for PD as well, including the rs908867, rs11030094, rs10501087, rs1157659, and rs1491850 SNPs (11, 14–16). We examined if these other *BDNF* variants are associated with either symptom severity or rate of symptom progression in idiopathic PD.

Without a validated biomarker of disease progression, symptom severity over time is still a key measurement that is difficult to interpret in the setting of confounding medication effects. Hence, the very early, unmedicated window starting after prompt diagnosis and ending with the requirement of symptomatic therapy is a critical time to examine progression. As such, we used data and samples from the Deprenyl And Tocopherol Antioxidative Therapy Of Parkinsonism (DATATOP) trial in a similar analysis to one performed previously for the rs6265 SNP (8), and we retrospectively examined subjects who were not receiving antiparkinsonian medications. We hypothesized that SNP genotype would alter the time a subject's PD symptoms progressed to the point of requiring symptomatic therapy with levodopa.

## Methods

We conducted a retrospective analysis on a subset of subjects who provided DNA from the DATATOP trial (17). We also queried publicly available gene expression data by tissue type in relation to variant status from The Genotype-Tissue Expression (GTEx) Project dataset (18). This research was determined by the Michigan State University Biomedical and Health Institutional Review Board not to meet the definition of human subjects research as defined by the U.S. Department of Health and Human Services regulations for the protection of human research subjects. All of the data from DATATOP are accessible through the United States National Institute of Neurological Disorders and Stroke (NINDS) or the GTEx online portal, and DATATOP genotype data are deposited as a Supplementary data file.

### DATATOP subjects and trial design

**Figure 1:**
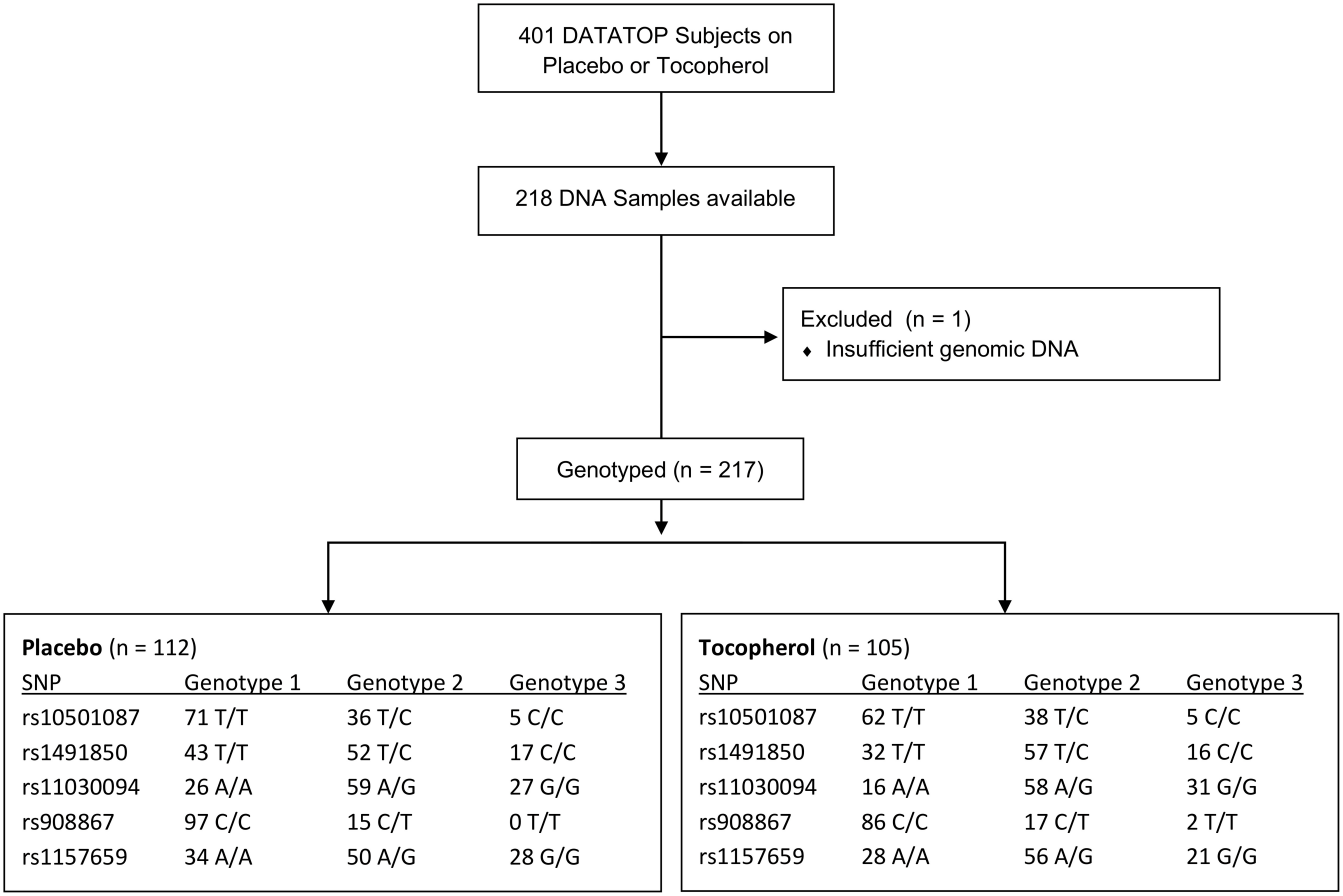
Flow diagram of sample acquisition and genotype group numbers. Diagrammed are the number of subjects in the DATATOP study, those who consented for DNA samples to be stored and the distribution of genotypes within each treatment arm for each genotype examined.

The DATATOP study included subjects with Hoehn and Yahr stages I or II with a duration of disease <5 years. All DATATOP subjects were diagnosed as having PD, with the DATATOP investigator maintaining ≥60% confidence in the PD diagnosis across all visits. The study was placebo-controlled and evaluated whether α-tocopherol, deprenyl or both could extend the time until requiring therapy with levodopa as a primary endpoint (17). DNA from subjects in both the placebo and α-tocopherol arms of the DATATOP trial was used in this study; subjects in the deprenyl arms were excluded due to the symptomatic effects reported. The decision to initiate levodopa therapy was determined by both the treating neurologist and the subject, considering the subject's functional disability based on the following factors: (a) threat to subject's employability, (b) threat to subject's ability to manage domestic and financial affairs, (c) an appreciable decline in the subject's handling of activities of daily living and (d) an appreciable worsening of gait or balance (17). The number of subjects genotyped by treatment arm is presented in Figure 1.

### SNP genotyping

DNA samples from DATATOP subjects were obtained from the biorepository at Indiana University and were genotyped for five *BDNF* SNPs that are not in the coding sequence, not known to alter BDNF protein structure but are associated with modest hippocampal or whole-brain atrophy, cognitive dysfunction or poorer clinical outcomes in early-stage PD subjects treated with levodopa monotherapy—viz., rs908867, rs11030094, rs10501087, rs1157659, and rs1491850 (11, 14–16). Genotype data for the *BDNF* rs6265 SNP in the DATATOP trial are publicly available (8). Genotyping was accomplished using the 5' exonuclease allelic discrimination Taqman assay (*n* = 217). Genotyping was performed with 20 ng of genomic DNA in a 25 μl reaction volume in duplicate using Taqman Genotyping Mastermix (Applied Biosystems, 4371353). Reactions were run in a Real-Time PCR instrument (7,500 Real Time PCR System, Applied Biosystems). Data were analyzed for genotype determination calls made by Taqman Genotyper software (Applied Biosystems). Subject genotype data are included for the research community (Supplementary data file).

### GTEx data

The data used for the analyses described in this manuscript were obtained from the GTEx Portal on 10/19/2022. An expression quantitative trait loci (eQTL) analysis was conducted to determine whether the examined SNPs—including the previously examined rs6265 SNP in the DATATOP cohort (8)—were associated with differences in *BDNF* transcript expression in brain tissues. Brain regions included in the query were: amygdala, anterior cingulate cortex (BA 24), caudate nucleus, cerebellar hemisphere, cerebellum, brain cortex, frontal cortex (BA 9), hippocampus, hypothalamus, nucleus accumbens, C1 cervical spinal cord and substantia nigra.

### Statistics

All statistical analyses were conducted using SAS v9.4 (Cary, NC). Subjects within the tocopherol and placebo treatment arms were pooled due to no significant differences in the primary endpoint reported at the conclusion of the DATATOP study (17). To test for significant deviations from Hardy Weinberg, the Pearson's chi-squared test was used to compare observed to expected genotype frequencies. Differences in baseline measures by genotype were examined. Independent *T*-tests and ANOVAs were used to test differences in means, and chi-square tests were used to test differences in proportions by *BDNF* SNP genotype. Kaplan-Meier curves were created to demonstrate the rate of levodopa treatment initiation over time for each genotype. Baseline age, sex, site, time since PD diagnosis and *BDNF* rs6265 genotype were entered as covariates in Cox proportional hazards models with time to needing levodopa since baseline as the outcome of interest. *BDNF* rs6265 genotype was included as a covariate due to a previous report of linkage disequilibria with the rs10501087 and rs1491850 SNPs (14) and a known effect of rs6265 on this study's outcome measures (8). The proportional hazards assumption of proportionality was tested by including a time-varying covariate in each model, defined as an interaction term between the predictor (genotype) and the event of time to needing levodopa. In the present analyses, the interaction term was not significant (*p* > 0.24) for each genotype except rs1491850, indicating that the assumption was not violated for these models. In the case of rs1491850, the interaction term was not significant when separate models were performed for time intervals <12 and >12 months, where 12 months is the observed median survival time (*p* = 0.99 and *p* = 0.19, respectively). The Cox proportional hazards results are presented separately for these time intervals for rs1491850. Secondary endpoints were estimates of annualized rates of change (calculated as the change from baseline to the time to needing dopaminergic therapy over the days in between the assessments multiplied by 365.25) in the following clinical characteristics: mini mental status score, Hamilton depression inventory, digit span forward, digit span backward, odd-man out test q15 + q19, odd-man out test q17 + q21, new dot test, verbal fluency, symbol digit modalities test, Purdue pegboard-right/left/both hand(s), Schwab/England activities of daily living (ADL) scale, Hoehn/Yahr stage and the Unified Parkinson Disease Rating Scale (UPDRS) total and parts 1 (mental), 2 (ADL) and 3 (motor) scores. Analysis of covariance was used to test significance of these secondary outcomes adjusting for the same covariates as described above plus the baseline value of the outcome. For the *BDNF* rs10501087 and rs908867 SNPs where the C/C and T/T genotypes, respectively, comprised <10 percent of the study population, these genotypes were pooled with heterozygous subjects and compared to the remaining genotype (i.e., homozygous for the more prevalent allele) to increase statistical power. Except for the GTEx analyses, statistical significance was set at *p* < 0.05 with the intention to interpret all analyses as requiring validation by a separate cohort. For the GTEx analyses, the query reports a T-statistic, and statistical significance was set at *p* < 0.01, again with the intention to interpret all results as requiring validation.

## Results

### Baseline characteristics

DATATOP subjects were genotyped successfully (Figure 1). Baseline characteristics were compared among the three combinations of allelic variants for each respective SNP (Supplementary Table 1). No significant differences were detected on any of the baseline characteristics for the *BDNF* rs10501087, rs908867, and rs1491850 SNPs. Differences for education (*p* < 0.01) and MMSE (*p* < 0.05) were detected at baseline for the rs1157659 and rs11030094 SNPs, respectively. Of note, DATATOP investigators maintained a ≥60% confidence in the PD diagnosis across all subjects and across all visits, as previously reported (8).

### Primary outcome measure—Time to initiation of dopaminergic therapy

**Figure 2:**
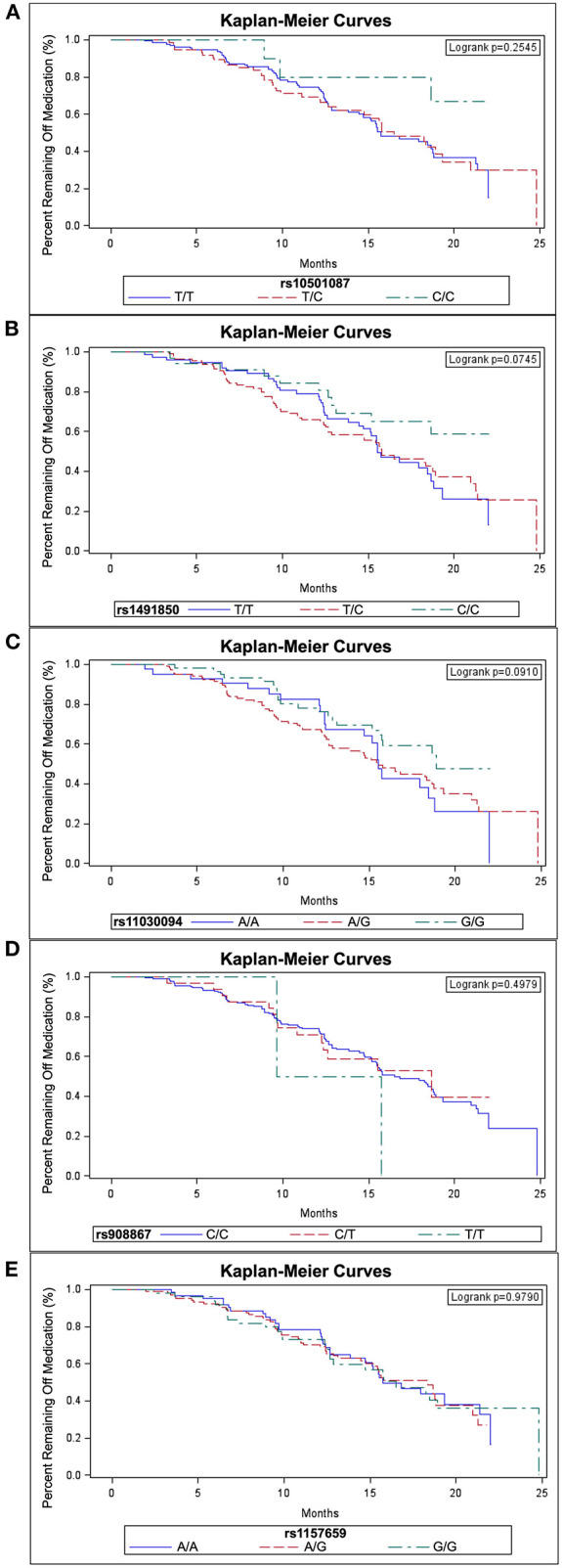
Survival curves for start of levodopa by *BDNF* SNP genotypes. Kaplan-Meier survival curves are depicted for the SNPs rs10501087 **(A)**, rs1491850 **(B)**, rs11030094 **(C)**, rs908867 **(D)** and rs1157659 **(E)** with subjects sorted by genotype.

The log-rank test based on a Kaplan-Meier analysis did not reveal an effect on time to start levodopa due to genotype for any SNP (Figure 2). However, when accounting for covariates using Cox proportional hazards models, survival analyses for the *BDNF* rs10501087 and rs1491850 SNPs revealed associations with delayed initiation of levodopa for subjects homozygous for the less prevalent allele (C/C for both) over the course of the study for rs10501087 and beyond month 12 for rs1491850 (Table 1). In addition, subjects homozygous for the more prevalent allele for the rs11030094 SNP were associated with delayed initiation as well (G/G, Table 1). In contrast, the rs908867 and rs1157659 SNPs did not alter time to initiate levodopa (Table 1).

**Table 1:** Survival analysis for time to needing dopaminergic therapy for placebo and α-tocopherol treated DATATOP subset with DNA by *BDNF* genotype.

	**Hazard ratio**	**95% CI**	***P-*value**
**rs10501087**
T/T (*n* = 133)	28.3	3.6–223.1	0.002*
T/C (*n* = 74)	7.6	1.9–29.8	0.004*
C/C (*n* = 10)	Referent		
C allele carrier vs. T/T	0.3	0.1–1.3	0.11
**rs908867**
T/T (*n* = 2)	2.8	0.6–13.7	0.21
C/T (*n* = 32)	1.2	0.6–2.4	0.53
C/C (*n* = 183)	Referent		
T allele carrier vs. C/C	1.4	0.7–2.6	0.35
**rs1157659**
G/G (*n* = 49)	1.1	0.6–2.1	0.74
A/G (*n* = 106)	0.9	0.5–1.6	0.75
A/A (*n* = 62)	Referent		
**rs11030094**
A/A (*n* = 42)	2.5	1.1–5.6	0.03*
A/G (*n* = 117)	2.0	1.1–3.8	0.03*
G/G (*n* = 58)	Referent		
**rs1491850**
**Time** **≤12 months**
T/T (*n* = 75)	1.7	0.5-6.0	0.40
T/C (*n* = 109)	2.0	0.7–5.9	0.21
C/C (*n* = 33)	Referent		
T allele carrier vs. C/C	2.0	0.7–5.8	0.22
**Time** **>12 months**
T/T (*n* = 75)	5.1	1.3–20.3	0.02*
T/C (*n* = 109)	3.0	0.9–+10.4	0.08
C/C (*n* = 33)	Referent		
T allele carrier vs. C/C	3.5	1.1–11.4	0.04*

Cox proportional hazards models adjusted for baseline age, sex, site, time since PD diagnosis and rs6265 genotype. Two separate models for rs1491850 at time intervals ≤ 12 and >12 months. CI, Confidence Interval.

* Denotes *p* < 0.05.

### Secondary outcome measures

All secondary outcome measures from the existing dataset were examined (Supplementary Tables 2–8). An effect of genotype was detected only for the odd-man out test q15 + q19 for the rs908867 SNP (*p* = 0.04, Supplementary Table 5). Otherwise, no effect of genotype was revealed by estimates of annualized rates of change for any of the other metrics, including mini mental status score, Hamilton depression inventory, digit spans forward and backward, new dot test, verbal fluency, symbol digit modalities test, Purdue pegboard for right, left and both hands, Schwab/England ADL scale, Hoehn/Yahr stage and UPDRS total, mental, motor and ADL scores (all *p* > 0.05, Supplementary Tables 2–8).

### SNPs associated with altered BDNF transcript expression

**Figure 3:**
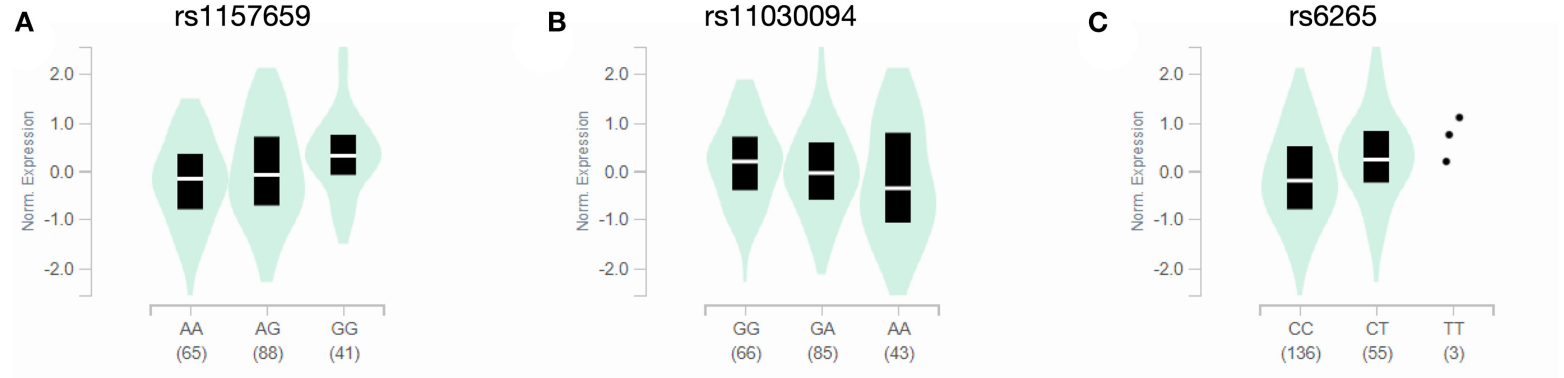
*BDNF* transcript expression in caudate nucleus by SNP. Expression quantitative trait loci (eQTL) violin plots from the GTEx dataset depict normative *BDNF* transcript expression in the caudate nucleus for the rs1157659 **(A)**, rs11030094 **(B)** and rs6265 **(C)** SNPs, grouped by genotype. The horizontal white line represents the median.

The GTEx dataset was queried to determine if any of the SNPs altered BDNF transcript expression across multiple sampled brain regions. Across all queried brain regions, gene expression associations only were observed in the caudate nucleus. The rs1157659 (*T* = 2.8; *p* = 0.0051), rs11030094 (*T* = −2.8; *p* = 0.0059) and rs6265 (*T* = 2.7; *p* = 0.0067) SNPs were associated with altered BDNF transcript expression (Figure 3).

## Discussion

A potential pharmacogenetic role for several *BDNF* SNPs is growing and may predict clinical outcomes in association with different treatment strategies (6, 11). Using an unmedicated cohort to remove the confound of effects of pharmacotherapy on clinical endpoints, the DATATOP and PPMI studies have been used to show a milder phenotype in association with subjects who are homozygous for the minor allele of the rs6265 SNP (8). As other *BDNF* SNPs have shown separate effects on clinical outcomes with levodopa monotherapy (6, 11), this study examined these in the DATATOP cohort as well and found that the rs10501087, rs1491850, and rs11030094 SNPs were associated with delayed initiation of symptomatic therapy for specific subject genotypes (C/C, C/C, and G/G, respectively) that correspond to 4.6, 15.2, and 26.7%, respectively, of this study's subjects.

The known neurobiology of the discussed *BDNF* SNPs is restricted primarily to the rs6265 SNP. Whereas this SNP is located in the protein-coding region of the gene and results in a Val66Met substitution in the protein's prodomain, the others are intronic. The rs6265 SNP results in reduced activity-dependent release of BDNF through reduced vesicular packaging (19). Despite not having a direct effect on protein structure, these intronic *BDNF* SNPs still are associated with brain atrophy (14, 16) and cognitive dysfunction (15). It may be that the other SNPs influence *BDNF* transcription by altering promotor region methylation (20), but their mechanisms have not been elucidated fully. Of note, the differences in time to levodopa initiation for the rs10501087, rs1491850, and rs11030094 SNPs in this study cannot be attributed to the rs6265 SNP since it was included in the statistical model as a covariate. Indeed, pharmacogenetic effects of these SNPs have been demonstrated when controlling for rs6265 genotype as a covariate in a large cohort of early-stage PD subjects (11), suggesting they may have distinct neurobiological effects. In addition, the transcript expression data analyzed in the present study suggest that some SNPs may alter *BDNF* expression in the caudate nucleus, and for the rs11030094 SNP, this may explain the association between the G/G genotype and prolonged time to initiation of levodopa. Of importance, SNPs in the gene *BDNF* in general may have quite disparate effects on PD progression (8), treatment strategy (11), risk for medication side effects (12, 13) and surgical candidacy (6, 21).

The DATATOP trial used the duration of time from diagnosis to when a patient requires symptomatic therapy as its primary outcome measure. This is a useful measure due to its clinical significance to neurologists and its meaning for patients. Its precision was strengthened from the use of prespecified criteria as a basis for discussion between the neurologist and the subject. This *post-hoc* study's finding of prolonged time from baseline to the decision to start symptomatic pharmacotherapy in association with specific rs10501087, rs1491850, and rs11030094 SNP genotypes likely reflects progression at least in early-stage disease. In contrast, these SNPs were not associated with effects on any of the secondary outcomes. Instead, secondary outcomes only detected a difference with the rs908867 SNP and without statistical adjustment for multiple comparisons, perhaps attributable to a type 1 error; indeed, no adjustment for multiple comparisons underscores the hypothesis-generating nature of this study, and all findings require validation. Of note, the number of C/C subjects for the rs10501087 or rs1491850 SNPs is quite low in this cohort, so their effects on secondary outcomes may be undetectable due to insufficient statistical power. Further, DATATOP subjects were enrolled with early-stage disease (with average disease duration of ~1.2 years), so genotype effect sizes may be reduced in this window (22).

Findings from this study add support to a potential role for variants in the gene *BDNF* in explaining PD heterogeneity. As for the specific SNPs examined in the present study, more research is needed to determine if a relationship exists with PD progression. This study used the DATATOP cohort as an exploratory analysis, and these findings should be considered as hypothesis-generating and requiring validation. A prospective clinical trial would provide higher quality evidence, though additional retrospective studies of existing cohorts (e.g., PPMI) may be conducted first to inform trial design. If validated, these findings may implicate other *BDNF* variants that have been explored much less than the rs6265 SNP and justify research into their neurobiology. Further, these SNPs may have pharmacogenetic effects (6, 11) that should be separated from any effect on PD progression, and this body of knowledge may inform clinical trial design.

## Conclusions

In an unmedicated cohort of early-stage PD subjects, specific genotypes for the *BDNF* rs10501087, rs1491850, and rs11030094 SNPs are associated with a prolonged time to the initiation of symptomatic therapy for PD motor symptoms, suggesting a slower rate of disease progression. A prospective clinical trial examining many *BDNF* SNPs is warranted to determine what effects, if any, exist on PD progression and for future use in clinical trial design.

## Data availability statement

The original contributions presented in the study are included in the article/Supplementary materials, further inquiries can be directed to the corresponding author/s.

## Ethics statement

The studies involving human participants were reviewed and approved by Michigan State University Biomedical and Health Institutional Review Board. The patients/participants provided their written informed consent to participate in this study.

## Author contributions

This research project was conceived by CS, DF, KP, and JG, organized by CS, KP, and DF and executed by CS, KP, AC-S, DF, CK, and JL. Statistical analyses were designed and executed by PA and were reviewed and critiqued by PA, CS, JG, and DF. The manuscript was first written by DF and CS, and it was reviewed and critiqued by all authors. All authors contributed to the article and approved the submitted version.

## Funding

This research was supported by grants from the Michael J. Fox Foundation and the Saint Mary's Foundation. Data used in the preparation of this article were obtained from the DATATOP Study. Funding for the DATATOP study was provided by the NIH/NINDS and the Michael J Fox Foundation for Parkinson's Research. The Genotype-Tissue Expression (GTEx) Project was supported by the Common Fund of the Office of the Director of the National Institutes of Health, and by NCI, NHGRI, NHLBI, NIDA, NIMH, and NINDS.

## Conflict of interest

Author CS receives research funding from the NIH, the Michael J. Fox Foundation and the Department of Defense, and she also receives income for reviewing for the NIH, the Michael J. Fox Foundation and the Weston Brain Institute. Author KP receives receives compensation for reviewing grants for the Michael J. Fox Foundation. Author JL receives research funding from the NIH and the Department of Defense, and he also receives income for reviewing for the NIH. Author JG receives research funding from the NIH, and he also receives income for reviewing for the NIH. The remaining authors declare that the research was conducted in the absence of any commercial or financial relationships that could be construed as a potential conflict of interest.

## Publisher's note

All claims expressed in this article are solely those of the authors and do not necessarily represent those of their affiliated organizations, or those of the publisher, the editors and the reviewers. Any product that may be evaluated in this article, or claim that may be made by its manufacturer, is not guaranteed or endorsed by the publisher.
