# Supplementary material for: BDNF rs10501087, rs1491850 and rs11030094 polymorphisms associated with delayed progression in early-stage Parkinson's disease
Source: Front Neurol. 2022 Nov 17;13:1053591. doi: 10.3389/fneur.2022.1053591 (PMC9713476; doi:10.3389/fneur.2022.1053591)
Supplement: Supplementary file 1 [file Table_1.docx]

**SUPPLEMENTARY TABLE S1**

Baseline Demographic and Clinical Characteristics of Placebo and α-Tocopherol Treated DATATOP Subset with DNA, by *BDNF* Genotype

rs10501087 rs908867

|  | T/T | T/C or C/C | p-value | C/C | C/T or T/T | p-value |
| --- | --- | --- | --- | --- | --- | --- |
| N | 133 | 84 |  | 183 | 34 |  |
| Males, % | 66.9 | 65.5 | 0.83 | 66.7 | 64.7 | 0.82 |
| Age, y | 60.6 (9.5) | 60.2 (7.4) | 0.73 | 60.3 (8.4) | 61.1 (10.5) | 0.64 |
| Education, y | 14.4 (3.7) | 14.3 (3.3) | 0.75 | 14.6 (3.4) | 13.2 (3.8) | 0.04 |
| Time since PD diagnosis, y | 1.2 (1) | 1.1 (1.1) | 0.32 | 1.2 (1.1) | 1.1 (1) | 0.75 |
| Mini mental status score | 29.1 (1.2) | 29.0 (1.4) | 0.47 | 29.1 (1.3) | 28.9 (1.3) | 0.46 |
| Hamilton depression inventory | 2.4 (2.8) | 2.6 (2.6) | 0.59 | 2.3 (2.7) | 3.1 (2.7) | 0.12 |
| Schwab/England ADL scale | 91.1 (6.8) | 92.1 (5.9) | 0.24 | 91.5 (6.1) | 91.1 (8.2) | 0.73 |
| UPDRS total | 25.2 (11.3) | 23.9 (10.9) | 0.38 | 24.8 (11.2) | 24.2 (11.1) | 0.78 |
| UPDRS motor | 17.1 (8.8) | 15.7 (8.2) | 0.27 | 16.7 (8.5) | 15.8 (8.9) | 0.61 |

Values are mean (standard deviation) unless otherwise noted.

rs1157659 rs11030094 rs1491850

|  | A/A | A/G | G/G | p-value | A/A | A/G | G/G | p-value | T/T | T/C | C/C | p-value |
| --- | --- | --- | --- | --- | --- | --- | --- | --- | --- | --- | --- | --- |
| N | 62 | 106 | 49 |  | 42 | 117 | 58 |  | 75 | 109 | 33 |  |
| Males, % | 61.3 | 67.0 | 71.4 | 0.52 | 69.1 | 63.3 | 70.7 | 0.57 | 68.0 | 64.2 | 69.7 | 0.79 |
| Age, y | 60.4 (9.0) | 60.9 (9.0) | 59.3 (8.0) | 0.57 | 62.6 (6.4) | 59.9 (9.8) | 59.9 (7.6) | 0.19 | 62.2 (8.5) | 59.5 (8.9) | 59.4 (8.1) | 0.09 |
| Education, y | 14.2 (3.4) | 13.9 (3.4) | 15.7 (3.7) | 0.01 | 14.3 (3.2) | 14.4 (3.7) | 14.4 (3.5) | 0.98 | 14.1 (3.7) | 14.5 (3.6) | 14.7 (3.0) | 0.72 |
| Time since PD diagnosis, y | 1.2 (1.1) | 1.3 (1.0) | 1.0 (1.0) | 0.39 | 1.1 (0.9) | 1.2 (1.0) | 1.2 (1.2) | 0.89 | 1.1 (1.0) | 1.2 (1.1) | 1.3 (1.2) | 0.82 |
| Mini mental status score | 29.0 (1.4) | 29.0 (1.3) | 29.1 (1.1) | 0.81 | 28.9 (1.3) | 29.2 (1.0) | 28.7 (1.7) | 0.03 | 29.0 (1.2) | 29.1 (1.2) | 29.0 (1.7) | 0.97 |
| Hamilton depression inventory | 2.4 (2.6) | 2.3 (2.8) | 2.8 (2.8) | 0.60 | 2.6 (2.5) | 2.3 (2.7) | 2.7 (2.9) | 0.54 | 2.8 (3.1) | 2.2 (2.4) | 2.7 (3.0) | 0.29 |
| Schwab/England ADL scale | 91.2 (7.5) | 91.1 (5.8) | 92.7 (6.3) | 0.34 | 90.5 (7.1) | 91.4 (6.8) | 92.4 (4.9) | 0.32 | 90.6 (7.7) | 92.0 (5.8) | 91.8 (5.2) | 0.37 |
| UPDRS total | 26.3 (11.5) | 24.3 (10.4) | 23.6 (12.2) | 0.39 | 26.0 (10.6) | 24.6 (11.8) | 23.9 (10.2) | 0.64 | 25.9 (11.4) | 23.8 (11.3) | 24.8 (10.0) | 0.45 |
| UPDRS motor | 18.0 (8.8) | 16.0 (8.2) | 15.9 (9.1) | 0.28 | 17.7 (8.3) | 16.4 (8.9) | 16.0 (8.2) | 0.61 | 17.4 (8.7) | 16.0 (8.9) | 16.3 (7.4) | 0.59 |

Values are mean (standard deviation) unless otherwise noted.
