# Supplementary material for: BDNF rs10501087, rs1491850 and rs11030094 polymorphisms associated with delayed progression in early-stage Parkinson's disease
Source: Front Neurol. 2022 Nov 17;13:1053591. doi: 10.3389/fneur.2022.1053591 (PMC9713476; doi:10.3389/fneur.2022.1053591)
Supplement: Supplementary file 2 [file Data_Sheet_1.docx]

**SUPPLEMENTARY TABLES S2-8**

Annualized Rates of Change Clinical Characteristics of Placebo and α-Tocopherol Treated DATATOP Subset with DNA, by *BDNF* Genotype

**Supplementary Table S2** rs10501087

|  | T/T  LSM (SE) | T/C  LSM (SE) | C/C  LSM (SE) | p-value |
| --- | --- | --- | --- | --- |
| Mini mental status score | -0.67 (0.84) | 1.18 (0.75) | -0.66 (1.61) | 0.22 |
| Hamilton depression inventory | 2.52 (2.40) | 3.58 (2.16) | 2.10 (4.77) | 0.92 |
| Digit span forward | 0.10 (0.51) | 0.14 (0.46) | 0.60 (1.00) | 0.89 |
| Digit span backward | 0.68 (0.66) | -0.30 (0.58) | -0.94 (1.27) | 0.59 |
| Odd-man out test q15+q19 | -0.16 (0.55) | -0.29 (0.49) | 0.57 (1.06) | 0.69 |
| Odd-man out test q17+q21 | 1.63 (0.67) | 0.52 (0.60) | 0.78 (1.31) | 0.61 |
| New dot test | -0.02 (0.39) | -0.68 (0.35) | 0.10 (0.77) | 0.35 |
| Verbal fluency | 8.31 (2.00) | 4.83 (1.80) | 3.87 (3.90) | 0.58 |
| Symbol digit modalities test | -2.74 (1.90) | 3.47 (1.71) | 3.33 (3.73) | 0.17 |
| Purdue pegboard – right hand | -1.82 (1.02) | 0.02 (0.93) | -0.14 (2.00) | 0.58 |
| Purdue pegboard – left hand | -0.82 (0.65) | -1.12 (0.60) | -0.20 (1.27) | 0.73 |
| Purdue pegboard – both hands | -2.19 (1.17) | -0.98 (1.05) | -2.08 (2.25) | 0.73 |
| Schwab/England ADL scale | -9.11 (3.30) | -9.13 (2.97) | -5.17 (6.44) | 0.81 |
| Hoehn/Yahr stage | 0.45 (0.21) | 0.35 (0.19) | 0.56 (0.40) | 0.83 |
| UPDRS total | 12.81 (3.80) | 15.50 (3.38) | 12.32 (7.32) | 0.83 |
| UPDRS mental | 0.49 (0.51) | 0.77 (0.46) | 0.77 (1.01) | 0.95 |
| UPDRS motor | 8.54 (2.81) | 9.76 (2.50) | 8.09 (5.41) | 0.92 |
| UPDRS ADL | 3.57 (1.16) | 4.80 (1.04) | 4.65 (2.25) | 0.82 |

Models are adjusted for site, sex, baseline age, duration of PD since diagnosis, rs6265 genotype, and baseline value of the outcome.

LSM=least mean square, SE=standard error

**Supplementary Table S3** rs10501087

|  | T/T  LSM (SE) | T/C or C/C  LSM (SE) | p-value |
| --- | --- | --- | --- |
| Mini mental status score | -0.77 (0.84) | 1.07 (0.74) | 0.20 |
| Hamilton depression inventory | 2.44 (2.38) | 3.49 (2.14) | 0.80 |
| Digit span forward | 0.12 (0.51) | 0.16 (0.46) | 0.96 |
| Digit span backward | 0.64 (0.65) | -0.33 (0.58) | 0.38 |
| Odd-man out test q15+q19 | -0.11 (0.54) | -0.23 (0.49) | 0.89 |
| Odd-man out test q17+q21 | 1.64 (0.67) | 0.53 (0.60) | 0.33 |
| New dot test | 0.02 (0.39) | -0.63 (0.35) | 0.33 |
| Verbal fluency | 8.27 (1.99) | 4.77 (1.78) | 0.31 |
| Symbol digit modalities test | -2.75 (1.89) | 3.46 (1.69) | 0.06 |
| Purdue pegboard – right hand | -1.83 (1.02) | 0.01 (0.92) | 0.30 |
| Purdue pegboard – left hand | -0.77 (0.65) | -1.06 (0.59) | 0.80 |
| Purdue pegboard – both hands | -2.25 (1.16) | -1.05 (1.04) | 0.55 |
| Schwab/England ADL scale | -8.90 (3.28) | -8.89 (2.94) | 0.99 |
| Hoehn/Yahr stage | 0.46 (0.20) | 0.37 (0.18) | 0.78 |
| UPDRS total | 12.63 (3.77) | 15.30 (3.34) | 0.68 |
| UPDRS mental | 0.49 (0.51) | 0.77 (0.46) | 0.75 |
| UPDRS motor | 8.44 (2.79) | 9.66 (2.47) | 0.80 |
| UPDRS ADL | 3.56 (1.15) | 4.79 (1.03) | 0.53 |

Models are adjusted for site, sex, baseline age, duration of PD since diagnosis, rs6265 genotype, and baseline value of the outcome.

LSM=least mean square, SE=standard error

**Supplementary Table S4** rs908867

|  | C/C  LSM (SE) | C/T  LSM (SE) | T/T  LSM (SE) | p-value |
| --- | --- | --- | --- | --- |
| Mini mental status score | 0.28 (0.35) | -0.22 (0.82) | 0.48 (3.05) | 0.84 |
| Hamilton depression inventory | 3.02 (1.00) | 3.15 (2.39) | -3.10 (8.82) | 0.78 |
| Digit span forward | 0.11 (0.22) | 0.35 (0.51) | 0.93 (1.89) | 0.83 |
| Digit span backward | 0.27 (0.27) | -0.74 (0.63) | -1.59 (2.37) | 0.25 |
| Odd-man out test q15+q19 | -0.33 (0.23) | 0.68 (0.53) | 2.24 (1.99) | 0.10 |
| Odd-man out test q17+q21 | 0.95 (0.28) | 1.66 (0.67) | 2.27 (2.46) | 0.53 |
| New dot test | -0.39 (0.16) | -0.04 (0.38) | 3.13 (2.09) | 0.17 |
| Verbal fluency | 6.25 (0.84) | 7.06 (1.98) | 16.16 (10.66) | 0.61 |
| Symbol digit modalities test | 0.82 (0.80) | -1.59 (1.88) | 9.78 (7.00) | 0.20 |
| Purdue pegboard – right hand | -0.77 (0.43) | -1.58 (1.01) | 2.27 (3.75) | 0.53 |
| Purdue pegboard – left hand | -0.90 (0.28) | -1.11 (0.65) | -1.03 (2.40) | 0.95 |
| Purdue pegboard – both hands | -1.71 (0.49) | -1.18 (1.14) | 2.22 (4.30) | 0.61 |
| Schwab/England ADL scale | -9.03 (1.37) | -7.19 (3.23) | -24.58 (12.18) | 0.37 |
| Hoehn/Yahr stage | 0.41 (0.09) | 0.38 (0.20) | 1.20 (0.75) | 0.57 |
| UPDRS total | 13.96 (1.58) | 14.26 (3.77) | 23.69 (13.74) | 0.78 |
| UPDRS mental | 0.56 (0.21) | 1.11 (0.51) | 1.71 (1.88) | 0.51 |
| UPDRS motor | 9.01 (1.17) | 9.53 (2.79) | 12.26 (10.17) | 0.94 |
| UPDRS ADL | 4.27 (0.48) | 3.64 (1.14) | 8.15 (4.24) | 0.56 |

Models are adjusted for site, sex, baseline age, duration of PD since diagnosis, rs6265 genotype, and baseline value of the outcome.

LSM=least mean square, SE=standard error

**Supplementary Table S5** rs908867

|  | C/C  LSM (SE) | C/T or T/T  LSM (SE) | p-value |
| --- | --- | --- | --- |
| Mini mental status score | 0.28 (0.35) | -0.17 (0.79) | 0.59 |
| Hamilton depression inventory | 3.04 (1.00) | 2.74 (2.30) | 0.90 |
| Digit span forward | 0.11 (0.21) | 0.38 (0.49) | 0.59 |
| Digit span backward | 0.27 (0.27) | -0.79 (0.61) | 0.10 |
| Odd-man out test q15+q19 | -0.33 (0.23) | 0.78 (0.51) | 0.04 |
| Odd-man out test q17+q21 | 0.94 (0.28) | 1.70 (0.64) | 0.27 |
| New dot test | -0.39 (0.17) | 0.07 (0.38) | 0.25 |
| Verbal fluency | 6.25 (0.84) | 7.38 (1.94) | 0.58 |
| Symbol digit modalities test | 0.79 (0.80) | -0.85 (1.83) | 0.40 |
| Purdue pegboard – right hand | -0.77 (0.43) | -1.33 (0.98) | 0.59 |
| Purdue pegboard – left hand | -0.90 (0.28) | -1.10 (0.62) | 0.75 |
| Purdue pegboard – both hands | -1.72 (0.49) | -0.97 (1.10) | 0.52 |
| Schwab/England ADL scale | -8.99 (1.38) | -8.24 (3.15) | 0.82 |
| Hoehn/Yahr stage | 0.41 (0.09) | 0.44 (0.20) | 0.90 |
| UPDRS total | 13.95 (1.58) | 14.86 (3.65) | 0.81 |
| UPDRS mental | 0.56 (0.21) | 1.15 (0.49) | 0.26 |
| UPDRS motor | 9.00 (1.17) | 9.70 (2.70) | 0.80 |
| UPDRS ADL | 4.26 (0.48) | 3.92 (1.10) | 0.77 |

Models are adjusted for site, sex, baseline age, duration of PD since diagnosis, rs6265 genotype, and baseline value of the outcome.

LSM=least mean square, SE=standard error

**Supplementary Table S6** rs1157659

|  | A/A  LSM (SE) | A/G  LSM (SE) | G/G  LSM (SE) | p-value |
| --- | --- | --- | --- | --- |
| Mini mental status score | 0.50 (0.62) | 0.26 (0.45) | -0.14 (0.66) | 0.78 |
| Hamilton depression inventory | 1.26 (1.79) | 3.51 (1.30) | 3.69 (1.87) | 0.52 |
| Digit span forward | -0.07 (0.38) | 0.11 (0.28) | 0.43 (0.40) | 0.66 |
| Digit span backward | 0.15 (0.49) | -0.12 (0.35) | 0.57 (0.52) | 0.54 |
| Odd-man out test q15+q19 | -0.24 (0.41) | -0.03 (0.30) | -0.41 (0.43) | 0.75 |
| Odd-man out test q17+q21 | 0.66 (0.50) | 0.90 (0.36) | 1.72 (0.52) | 0.31 |
| New dot test | -0.74 (0.29) | -0.15 (0.21) | -0.27 (0.32) | 0.23 |
| Verbal fluency | 3.54 (1.50) | 7.30 (1.08) | 7.45 (1.59) | 0.08 |
| Symbol digit modalities test | -0.60 (1.44) | 1.15 (1.04) | 0.55 (1.50) | 0.58 |
| Purdue pegboard – right hand | -0.54 (0.76) | -0.51 (0.56) | -1.84 (0.81) | 0.37 |
| Purdue pegboard – left hand | -1.29 (0.48) | -0.41 (0.35) | -1.59 (0.51) | 0.10 |
| Purdue pegboard – both hands | -1.37 (0.86) | -0.89 (0.63) | -3.31 (0.92) | 0.10 |
| Schwab/England ADL scale | -7.69 (2.46) | -9.17 (1.79) | -9.52 (2.57) | 0.85 |
| Hoehn/Yahr stage | 0.26 (0.15) | 0.38 (0.11) | 0.61 (0.16) | 0.27 |
| UPDRS total | 12.86 (2.82) | 13.88 (2.07) | 15.60 (2.93) | 0.79 |
| UPDRS mental | 0.72 (0.38) | 0.80 (0.28) | 0.26 (0.40) | 0.53 |
| UPDRS motor | 8.87 (2.08) | 8.58 (1.53) | 10.29 (2.17) | 0.81 |
| UPDRS ADL | 3.30 (0.86) | 4.42 (0.62) | 4.70 (0.90) | 0.46 |

Models are adjusted for site, sex, baseline age, duration of PD since diagnosis, rs6265 genotype, and baseline value of the outcome.

LSM=least mean square, SE=standard error

**Supplementary Table S7** rs11030094

|  | A/A  LSM (SE) | A/G  LSM (SE) | G/G  LSM (SE) | p-value |
| --- | --- | --- | --- | --- |
| Mini mental status score | 0.27 (0.79) | 0.53 (0.44) | -0.33 (0.62) | 0.55 |
| Hamilton depression inventory | 4.12 (2.28) | 3.27 (1.25) | 2.04 (1.77) | 0.77 |
| Digit span forward | -0.13 (0.49) | 0.19 (0.27) | 0.18 (0.38) | 0.82 |
| Digit span backward | 1.06 (0.61) | 0.03 (0.34) | -0.12 (0.48) | 0.24 |
| Odd-man out test q15+q19 | -0.06 (0.52) | -0.34 (0.29) | 0.07 (0.40) | 0.67 |
| Odd-man out test q17+q21 | 1.16 (0.64) | 0.81 (0.35) | 1.43 (0.49) | 0.57 |
| New dot test | -0.57 (0.38) | -0.48 (0.21) | 0.06 (0.29) | 0.30 |
| Verbal fluency | 4.30 (1.95) | 6.42 (1.04) | 7.23 (1.49) | 0.48 |
| Symbol digit modalities test | 0.85 (1.83) | 0.20 (1.00) | 1.09 (1.42) | 0.85 |
| Purdue pegboard – right hand | -2.80 (0.97) | -0.62 (0.53) | -0.46 (0.74) | 0.09 |
| Purdue pegboard – left hand | -1.45 (0.62) | -0.97 (0.34) | -0.62 (0.48) | 0.58 |
| Purdue pegboard – both hands | -3.26 (1.11) | -1.65 (0.61) | -0.89 (0.86) | 0.23 |
| Schwab/England ADL scale | -12.61 (3.13) | -8.44 (1.71) | -8.13 (2.43) | 0.43 |
| Hoehn/Yahr stage | 0.60 (0.20) | 0.38 (0.11) | 0.39 (0.15) | 0.57 |
| UPDRS total | 15.41 (3.60) | 14.1 (1.95) | 13.44 (2.84) | 0.91 |
| UPDRS mental | -0.43 (0.48) | 0.72 (0.26) | 0.94 (0.37) | 0.05 |
| UPDRS motor | 12.13 (2.65) | 9.05 (1.44) | 7.91 (2.09) | 0.44 |
| UPDRS ADL | 4.06 (1.10) | 4.52 (0.60) | 3.74 (0.85) | 0.74 |

Models are adjusted for site, sex, baseline age, duration of PD since diagnosis, rs6265 genotype, and baseline value of the outcome.

LSM=least mean square, SE=standard error

**Supplementary Table S8** rs1491850

|  | T/T  LSM (SE) | T/C  LSM (SE) | C/C  LSM (SE) | p-value |
| --- | --- | --- | --- | --- |
| Mini mental status score | -0.01 (0.67) | 0.48 (0.43) | -0.30 (0.82) | 0.62 |
| Hamilton depression inventory | 3.65 (1.93) | 3.13 (1.24) | 1.74 (2.36) | 0.83 |
| Digit span forward | 0.13 (0.41) | 0.09 (0.27) | 0.34 (0.50) | 0.91 |
| Digit span backward | 0.14 (0.52) | 0.05 (0.34) | 0.29 (0.65) | 0.94 |
| Odd-man out test q15+q19 | 0.49 (0.43) | -0.58 (0.28) | 0.22 (0.53) | 0.07 |
| Odd-man out test q17+q21 | 1.50 (0.54) | 0.93 (0.35) | 0.83 (0.66) | 0.64 |
| New dot test | 0.01 (0.32) | -0.59 (0.20) | 0.07 (0.38) | 0.13 |
| Verbal fluency | 5.63 (1.62) | 7.25 (1.03) | 4.76 (1.96) | 0.43 |
| Symbol digit modalities test | -0.38 (1.54) | 1.28 (0.99) | -0.44 (1.88) | 0.53 |
| Purdue pegboard – right hand | -2.18 (0.82) | -0.56 (0.53) | -0.07 (1.00) | 0.17 |
| Purdue pegboard – left hand | -1.57 (0.52) | -0.60 (0.34) | -1.07 (0.64) | 0.26 |
| Purdue pegboard – both hands | -2.49 (0.94) | -1.16 (0.60) | -1.90 (1.13) | 0.45 |
| Schwab/England ADL scale | -11.82 (2.63) | -7.69 (1.70) | -8.80 (3.22) | 0.40 |
| Hoehn/Yahr stage | 0.68 (0.16) | 0.30 (0.11) | 0.39 (0.20) | 0.14 |
| UPDRS total | 14.96 (3.05) | 14.26 (1.95) | 12.32 (3.73) | 0.87 |
| UPDRS mental | 0.06 (0.41) | 0.85 (0.26) | 0.72 (0.50) | 0.26 |
| UPDRS motor | 11.21 (2.25) | 8.56 (1.44) | 8.04 (2.75) | 0.56 |
| UPDRS ADL | 4.16 (0.93) | 4.57 (0.59) | 3.18 (1.13) | 0.55 |

Models are adjusted for site, sex, baseline age, duration of PD since diagnosis, rs6265 genotype, and baseline value of the outcome.

LSM=least mean square, SE=standard error
